# Accuracy of maxillary full‐arch digital impressions of tooth and implant models made by two intraoral scanners

**DOI:** 10.1002/cre2.857

**Published:** 2024-03-03

**Authors:** Hakimeh Siadat, Foujan Chitsaz, Somayeh Zeighami, Alireza Esmaeilzadeh

**Affiliations:** ^1^ Department of Prosthodontics, School of Dentistry, Dental Research Center, Dentistry Research Institute Tehran University of Medical Sciences Tehran Iran; ^2^ School of Mechanical and Manufacturing Engineering University of New South Wales Sydney New South Wales Australia; ^3^ Department of Prosthodontics, School of Dentistry Arak University of Medical Sciences Arak Iran

**Keywords:** dental implants, dental impression technique, dimensional measurement accuracy

## Abstract

**Objectives:**

Limited studies are available on the accuracy of intraoral scanners (IOSs) for full‐arch implant and tooth models. This study aimed to assess the accuracy of maxillary full‐arch digital impressions of tooth and implant models made by two IOSs.

**Materials and Methods:**

This in vitro, experimental study was conducted on two maxillary dentiform models: one with six prepared natural teeth and the other with six implants at the site of canine, first premolar, and first molar teeth, bilaterally. A highly accurate industrial scanner was used for actual measurements on the models that served as the reference scan. TS (Trios3) and CO (CEREC Omnicam) IOSs were then used to scan each model 10 times according to the manufacturer's instructions. All scans were saved in STL format. The GOM Inspect software was used according to the best‐fit algorithm to compare the accuracy of measurements in the groups with the reference scan. The trueness and precision were calculated. Statistical analyses were carried out using SPSS by one‐way analysis of variance and *t*‐test (*α* = .05).

**Results:**

TS showed a significantly higher trueness than CO for both tooth and implant models (*p* < .05). TS also revealed significantly higher precision than CO for the tooth model; however, the difference in precision for the implant model was not significant between the two IOSs (*p* > .05).

**Conclusions:**

TS showed higher accuracy than CO in both tooth and implant models.

## 1 INTRODUCTION

Precise transfer of the three‐dimensional (3D) position of implants to the master cast is a critical step to achieve passive fit in implant‐supported fixed partial dentures (IFPDs). Low precision of impressions and errors in manual steps of prosthetic fabrication can result in misfit and subsequent biological, mechanical, and technical complications. Researchers have attempted to quantify misfit, and reach a consensus regarding its acceptable level; however, their opinions on this topic are highly controversial (Di Fiore et al., 2019). According to Branemark, misfit should not exceed 10 µm (Branemark, 1983), (Adell, 1985), while Jemt (1991) showed that misfits up to 150 µm are acceptable. The significance of optimal fit has been well documented in the clinical setting (Chochlidakis et al., 2016). Precision and trueness are also calculated to assess the accuracy of impressions. Trueness refers to the difference between the actual values and those yielded by scanning, while precision is defined as the difference between repeated measurements made on the same model.

Polyether and polyvinyl siloxane have excellent dimensional stability and accuracy, and have been successfully used for IFPDs and FPDs. However, factors such as temperature change, time interval between impression making and pouring, wettability of dental stone, and disinfection process may cause distortion of impression materials and compromise the accuracy of impressions. Also, application of die hardener and spacer as well as the laboratory phases of restoration fabrication can cause dimensional changes and adversely affect the fit of final restoration (Chochlidakis et al., 2016). At present, conventional impressions with different techniques and materials are still a common practice; however, due to advances in digital impression techniques, they are gradually replacing the conventional impression techniques. The main reason behind the increasing popularity of digital impressions is their comparable accuracy to the conventional impressions (Di Fiore et al., 2019). Digital impressions have numerous advantages for IFPDs and FPDs, such as elimination of laboratory steps that could cause misfit, faster preparation, and lower patient discomfort (Chochlidakis et al., 2016).

A number of studies have documented the comparable accuracy of digital and conventional impression techniques for single crowns (Boeddinghaus et al., 2015; Zarauz et al., 2016) and single‐implant restorations with no significant difference in marginal adaptation of restorations (Lee et al., 2015). However, a clinical study compared the digital and conventional impression techniques and showed that using an intraoral scanner (IOS) regardless of finish line location improved the adaptation of restorations (Koulivand et al., 2020).

However, controversy still exists regarding full‐arch digital impressions. Some authors reported comparable accuracy of digital and conventional full‐arch impressions (Ender & Mehl, 2011; Papaspyridakos et al., 2016; Patzelt et al., 2014), while some others showed lower accuracy of digital compared with conventional impressions (Ender & Mehl, 2013, 2015; Giménez et al., 2014, 2015a, 2015b). Controversy in the results may be due to the use of different methods or models for assessment of the accuracy of IOSs. Some studies used a full‐arch model of the maxilla with all teeth present, while some others used a model of mandible with five or six implants (Di Fiore et al., 2019). Ender and Mehl (2011, 2015) and Ender, Attin, et al. (2016) reported that the accuracy of full‐arch digital impressions was lower than that of silicone impressions and higher than that of polyether and irreversible hydrocolloid impressions. Another study demonstrated higher accuracy of digital impressions for one quadrant compared with silicone impressions (Ender, Zimmermann, et al., 2016). In full‐arch digital impressions, a tiny inaccuracy can lead to misfit; however, in sextant impressions, there is no evidence to support the effect of scanning strategy on impression accuracy (Pesce et al., 2018). The effect of depth of insertion and angulation of implants on the accuracy of intraoral scans has also been studied (Giménez et al. 2014, 2015a, 2015b; Gimenez‐Gonzalez et al., 2017). The accuracy of conventional implant impressions decreases with an increase in the number of implants, due to the deformation of impression during removal (Sorrentino et al., 2010). This statement is also true for digital impressions due to the need for recording of a greater pile of data. The field of view effect is the most important reason for low accuracy of IOSs reported in the literature. Evidence shows that the larger the area scanned by IOS, the greater the errors of digital impressions could be; such large errors are due to the cumulative effects of scanning errors (Zimmermann et al., 2015). Also, different IOSs may have different levels of accuracy (Takeuchi et al., 2018). TS and CO are two commonly used IOSs. However, the results regarding their accuracy have been controversial depending on the scanning strategy, scanned area (full‐arch/sextant), and implant/tooth models (Di Fiore et al., 2019; Medina‐Sotomayor et al., 2018; Mennito et al., 2018; Nedelcu et al., 2018; Renne et al., 2017; Vandeweghe et al., 2017). All the available studies comparing the accuracy of IOSs for full‐arch models reported higher accuracy of TS than CO (Di Fiore et al., 2019; Medina‐Sotomayor et al., 2018; Mennito et al., 2018; Nedelcu et al., 2018; Renne et al., 2017; Vandeweghe et al., 2017); however, both of them are among the most accurate IOSs (Di Fiore et al., 2019; Diker & Tak, 2020; Imburgia et al., 2017; Joda & Brägger, 2014; Lee & Gallucci, 2013; Mangano et al., 2019).

Considering the increasing use of IOSs and decreasing use of impression materials, this study aimed to assess the accuracy of maxillary full‐arch digital impressions of tooth and implant models made with two IOSs. The null hypothesis of the study was that the accuracy of the two IOSs would be equal.

## 2 MATERIALS AND METHODS

The study was approved by the ethics committee of Tehran University of Medical Sciences (IR.TUMS.DENTISTRY.REC.1398.116). Informed consent was obtained from patients whose extracted teeth were used in this study. The study was performed in line with the principles of the Declaration of Helsinki.

To standardize the samples, this in vitro, experimental study was conducted on two maxillary dentiforms (Hasban Mandegar, Tehran, Iran). The acrylic canine, first premolar, and first molar teeth of the model were removed bilaterally, and implants were placed at the empty sites in one dentiform, while extracted natural teeth were placed at the empty sites in the other dentiform.

The sample size was calculated to be 9 in each of the four groups, according to a study by Medina‐Sotomayor et al. (2018) assuming *α *= .05, *β *= .2, effect size = 0.6, and mean standard deviation of 22.34 for trueness using one‐way analysis of variance feature of PASS 11. To ensure the accuracy of the results, 10 specimens were used in each group. The required sample size for precision was calculated to be 6 (Medina‐Sotomayor et al., 2018).

### 2.1 Preparation of implant model

**Figure 1:**
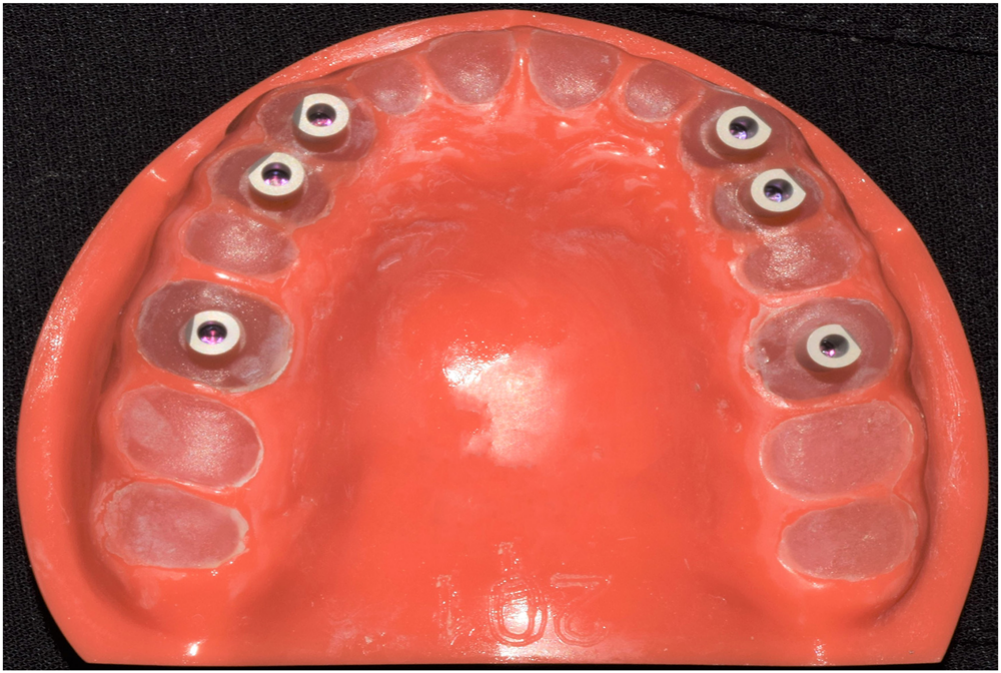
Implant model after placement of scan bodies.

In the first dentiform model, six bone‐level implant analogs with 12 mm length (Straumann RC) were bilaterally placed at the site of extracted teeth (canine, first premolar, and first molar teeth) along their longitudinal axis, and fixed in place with auto‐polymerizing acrylic resin (Hard Denture, Chairside Reline Material; GC USA) such that the implant analog margin was placed subgingivally by 1 mm. The posterior implant analogs were placed parallel to each other, and the canine implant analogs were also parallel to each other bilaterally (Figure 1).

### 2.2 Preparation of tooth model

**Figure 2:**
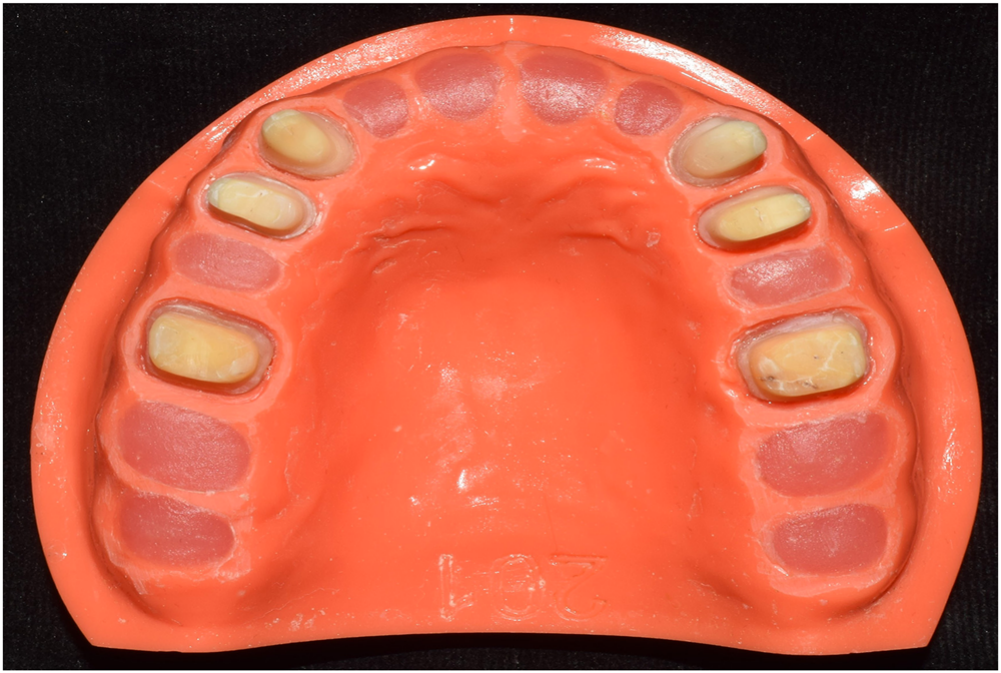
Final dental model.

Maxillary left and right canine, first premolar, and first molar teeth (1 tooth from each type) extracted due to periodontal disease or orthodontic treatment were collected, rinsed with water, disinfected with 5% sodium hypochlorite, and stored in water until use. Before mounting in a dentiform model, the teeth were mounted in putty impression material, and received a full‐coverage preparation for an all‐ceramic restoration using a mesiodistal and buccopalatal index. The incisal reduction for anterior teeth and occlusal reduction for posterior teeth was 2 mm, and the facial and lingual reduction was 1 mm. The teeth also received a radial shoulder finish line with 1 mm depth. The optimal taper ranged from 10° for canine teeth to 22° for molar teeth. Six prepared natural tooth crowns were mounted at the site of extracted teeth bilaterally (canine, first premolar, and first molar teeth) along the longitudinal axis of extracted teeth using auto‐polymerizing acrylic resin (Hard Denture, Chairside Reline Material; GC USA). The finish line of prepared teeth was at the level of gingiva. The posterior teeth at each side were positioned parallel to each other and the canine teeth were also parallel. Corrections were then made by removing the undercuts (Figure 2).

### 2.3 Scanning and superimposition

**Figure 3:**
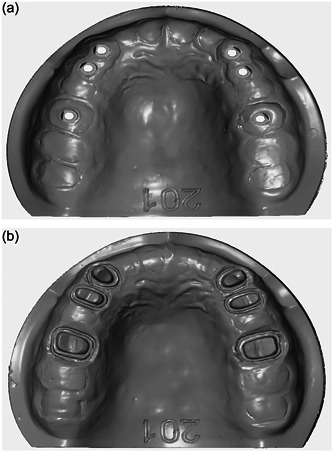
(a) Scanning of implant model with an industrial scanner to serve as a reference model. (b) Scanning of the tooth model with an industrial scanner to serve as a reference model.

A highly accurate industrial scanner (ATOS Core 80; GOM) was used for actual measurements of the reference models. The obtained scan files served as the reference for the measurements (Figure 3).

To scan the implant model, scan bodies (Straumann® CARES® Mono Scan body) were hand tightened on implants according to the manufacturer's instructions (max. 15 N cm). The groove on scan bodies was placed in the buccal to standardize the conditions for all scans and enhance their superimposition. Each model was scanned by each of the TS (Trios3, 3Shape) and CO (CEREC Omnicam, Dentsply Sirona Inc.) IOSs 10 times by an experienced operator (after performing 16 practice scans according to the learning curve). The scanning strategy was standard as instructed by the manufacturers.

**Figure 4:**
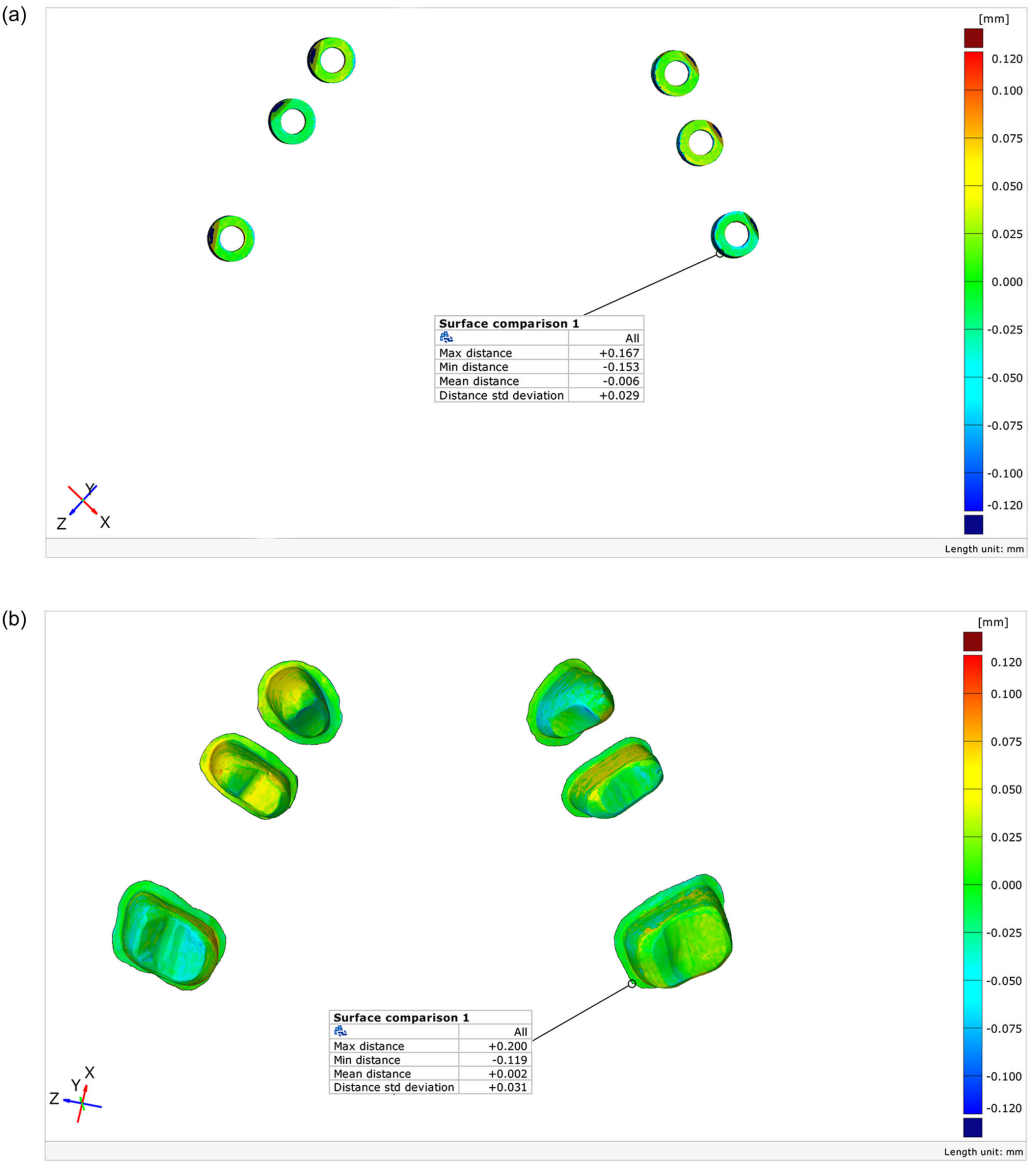
(a) Report of GOM Inspect software regarding trueness and precision. (b) Report of GOM Inspect software regarding trueness and precision.

To compare the accuracy of each group with the reference model, the GOM Inspect diagnostic software with the best‐fit algorithm was used (Diker & Tak, 2020). For this purpose, the two full‐arch scan files were selected. The reference file was defined as CAD, and the next file was defined as Mesh. Next, the prealignment feature was selected to superimpose the files. The areas around implants and teeth were omitted such that only the position of teeth, implants, and scan bodies relative to each other remained. In the next step, the Mesh file was selected and subjected to local best fit compared to the CAD file. Finally, the report was obtained by commanding surface comparison on CAD. The GOM software revealed the existing differences, whether positive (expansion) or negative (shrinkage), in micrometer scale (Figure 4). To assess the accuracy, trueness (the difference between actual values and those yielded by scanning) and precision (the difference between repeated measurements made on the same model) were calculated.

Statistical analyses were carried out using SPSS version 22 (IBM Corp.). Also, trueness and precision of different groups were compared by one‐way analysis of variance and *t*‐test (*α* = .05).

## 3 RESULTS

Table 1 shows the trueness of the two IOSs for the tooth and implant models. As shown, in both tooth (*p* < .0001) and implant (*p* < .0001) models, the trueness for TS was significantly higher than that of CO.

**Table 1:** Trueness of the two intraoral scanner for the tooth and implant models in micrometers *(n* = 10).

Model	Scanner	Mean (µm)	SD (µm)	SE (µm)	Minimum (µm)	Maximum (µm)	Independent samples test
Tooth	CO	46.2000	7.40570	2.34189	35.00	53.00	*p* = .000
	TS	25.7000	2.71006	0.85700	23.00	31.00	
	Total	35.9500	11.83427	2.64622	23.00	53.00	
Implant	CO	126.0000	40.08047	12.67456	97.3282	154.6718	*p* = .000
	TS	45.0000	25.33772	8.01249	26.8745	63.1255	
	Total	85.5000	52.83589	11.81446	60.7720	110.2280	

Abbreviations: CO, CEREC Omnicam; TS, Trios3.

Table 2 presents the precision of the two IOSs for the tooth and implant models. As indicated, the precision in the tooth model in TS was significantly higher than that of CO (*p* < .0001). For the implant model, the precision of TS was higher than that of CO, but the difference was not statistically significant (*p* > .05).

**Table 2:** Precision of the two intraoral scanner for the tooth and implant models in micrometers (*n* = 45).

Model	Scanner	Mean (µm)	SD (µm)	Minimum (µm)	Maximum (µm)	Paired samples test
Tooth	CO	105.3556	20.07051	41.00	147.00	*p* = .000
	TS	55.4222	22.07991	25.00	129.00	
Implant	CO	208.1778	38.52348	121.00	326.00	*p* = .852
	TS	206.4444	55.67297	113.00	326.00	

Abbreviations: CO, CEREC Omnicam; TS, Trios3.

## 4 DISCUSSION

The results showed that the trueness for both tooth and implant models and precision in the tooth model were significantly higher for TS. For the implant model, the precision of TS was insignificantly higher than that of CO. Mangano et al. (2019). compared the accuracy of TS and CO in a completely edentulous model of maxilla reconstructed with six implants. They reported that the trueness and precision of TS were higher than the corresponding values for CO. Vandeweghe et al. (2017) evaluated a maxillary model with six external‐connection implants and reported that the trueness and precision of Trios were lower than those of CO. The same results were reported by Imburgia et al. (2017) for an edentulous model of maxilla with six implants, which confirms the present results.

Digital implant impressions can be challenging. Although a number of studies have indicated that scanning of one implant unit can be performed with high predictability (Abdel‐Azim et al., 2014; Joda & Brägger, 2014; Lee & Gallucci, 2013), some limitations exist regarding full‐arch intraoral scans. Several studies evaluated full‐arch digital impressions on models simulating the fabrication of an implant‐supported suprastructure for an edentulous jaw. They reported that digital impressions had trueness and precision equal (Papaspyridakos et al., 2016) or higher (Abdel‐Azim et al., 2014; Amin et al., 2017; Baghani et al., 2021; Menini et al., 2018) than those of conventional impressions. Zhang et al. (2021) in their systematic review indicated a trueness of 7.6–731.7 µm and precision of 15.2–204.2 µm for digital implant impressions. In the present study, the mean trueness for the implant model scanned with TS was 45.00 µm, which was comparable to the trueness of Trios Color Cart reported by Bilmenoglu et al. (2020), which was 40.3 µm, and the value of 32 µm reported by di Fiore et al. (2019) in their study on an edentulous model of mandible with six implants. This value was 71.6 µm in a study by Mangano et al. (2019) and 67.2 µm for an edentulous model of maxilla with six implants in a study by Imburgia et al. (2017).

The mean trueness for implant models scanned with CO was 126.00 µm in the present study, which was higher than the value reported by Imburgia et al. (2017), which was 66.4 µm. In the present study, the mean trueness for digital dental impressions scanned with CO was 46.2 µm, which was higher than the value (19.13 µm) reported by Passos et al. (2019). This difference may be due to interindividual differences of the operators, fewer teeth, and using prepared teeth in the tooth model, and differences in scanning strategy of the present study.

The mean precision of digital impressions for the implant model was 206.4 µm by TS and 208.1 µm by CO in the present study, which were not significantly different. This finding was similar to the results of Mangano et al. (2019) and Imburgia et al. (2017) for digital full‐arch impressions of the maxilla with six implants.

In comparison of the two IOSs, TS showed superior performance compared with CO for full‐arch digital impressions, which may be due to its unique design. CO, unlike TS, does not have an inbuilt heater. The presence of an inbuilt heater in TS prevents fogging of the mirror head of the scanner in the oral environment. In CO, the scanner heater is located on the cart, and warms the scanner while placed on the cart. Although this amount of heat is often sufficient to prevent fogging while scanning a simple quadrant, it may decrease the accuracy in full‐arch scanning especially by novice operators.

Stability of the surface to be scanned is a challenge in digital impressions. The form of mucosa may change depending on the type of jaw movement, which compromises the scanning process (Andriessen et al., 2014). Also, in edentulous arches, a limited number of reference points between the scan bodies is another challenge which prevents correct stitching of images, or results in interpretation of some parts of the scan as excess data (Mizumoto & Yilmaz, 2018). Another challenge in edentulous arches reconstructed with implants is the use of several look‐alike scan bodies, which may complicate their differentiation from each other and identification of their correct location in dental arch for the IOS (Vandeweghe et al., 2017). Furthermore, the shape, height, and angulation of scan bodies can affect the final results. Gómez‐Polo et al. (2022) evaluated two maxillary casts reconstructed with six implants. The first cast had six completely parallel analogs while the second cast had six analogs with maximally 30° divergence relative to each other. They used three scan bodies with 3, 6, and 10 mm height. The results revealed that implant angulation and clinical height of scan bodies affected the accuracy of scans. The shortest scan body had the lowest scanning accuracy in both casts. Moslemion et al. (2020) demonstrated that the shape and type of scan bodies used for maxillary reconstruction with an all‐on‐four design affected the accuracy of digital impressions. In the present study, Straumann scan bodies were used, which have acceptable accuracy for digital scanning based on their height. On the other hand, considering the difficult scanning of the surface of scan bodies in the implant model, compared with the tooth model, the obtained findings seem logical. The present results were in agreement with the findings of previous studies regarding difficult digital scanning of edentulous arches and negative effects of interferences of anatomical irregularities and large areas between anatomical landmarks with the best fit process in use of IOSs (Andriessen et al., 2014; Iturrate et al., 2019; Kim et al., 2017; Vandeweghe et al., 2017).

In vitro studies can be of great value to limit the effect of confounding variables on the results. Due to the differences between the oral environment and in vitro conditions, generalization of results to the clinical setting should be done with caution. Different substrates such as amalgam, composite resin, metal, ceramic, enamel, and dentin may be present in the oral cavity which have different optical and light reflectance properties compared with dentiforms or gypsum casts. Oral tissues are not ideal for light reflectance because their translucency compromises the process of image stitching. The oral environment has variable temperature and moisture conditions. Moreover, the oral tissues are mobile. The presence of saliva, blood, and gingival crevicular fluid can also affect the accuracy of digital scans. The intraoral space may not be sufficient for placement and movement of scanner head in posterior regions, and can also limit the vision. The dentiforms used in the present study, have a shiny surface and highly reflect light, but they do not have mobile mucosa like oral mucosa, which is considered a drawback. Ambient light conditions were found to affect the accuracy (Revilla‐León et al., 2020; Wesemann et al., 2021) and scanning time (Wesemann et al., 2021) of IOSs. In the current study, all samples were scanned under room light; thus, the ambient light was not a confounding factor. Another limitation was the fact that only the recommended scanning strategy was adopted.

The effects of other variables such as the distance between the scanned teeth or implants, length of scanned arch, and shape of scan bodies on the accuracy of digital impressions are among other interesting topics for further research.

## 5 CONCLUSION

Within the limitations of this in vitro study, the results showed higher trueness and precision (accuracy) of TS for the tooth and implant models.

## AUTHOR CONTRIBUTIONS

*Study concept and design and study supervision*: Somayeh Zeighami and Hakimeh Siadat. *Acquisition of data and Statistical analysis*: Somayeh Zeighami and Alireza Esmaeilzadeh.  *Analysis and interpretation of data*: Somayeh Zeighami, Hakimeh Siadat, and Alireza Esmaeilzadeh. *Drafting of the manuscript*: Somayeh Zeighami and Foujan Chitsaz. *Critical revision of the manuscript for important intellectual content*: Somayeh Zeighami, Hakimeh Siadat, and Foujan Chitsaz. *Administrative, technical, and material support*: Somayeh Zeighami.

## CONFLICT OF INTEREST STATEMENT

The authors declare no conflict of interest.

## ETHICS STATEMENT

The study was approved by the ethics committee of Tehran University of Medical Sciences (IR.TUMS.DENTISTRY.REC.1398.116). Informed consent was obtained from patients whose extracted teeth were used in this study. All methods were carried out in accordance with relevant guidelines and regulations.

## Data Availability

The data used to support the findings of this study were supplied by the corresponding author under license and data will be available on request. Requests for access to these data should be made to the corresponding author.
